# Modular Organization of the ESX-5 Secretion System in *Mycobacterium tuberculosis*

**DOI:** 10.3389/fcimb.2016.00049

**Published:** 2016-05-02

**Authors:** Swati Shah, Volker Briken

**Affiliations:** Department of Cell Biology and Molecular Genetics, University of MarylandCollege Park, MD, USA

**Keywords:** *Mycobacterium tuberculosis*, ESX-1, ESX-5, PE/PPE proteins, type 7 secretion systems, protein secretion

## Abstract

Mycobacteria utilize type VII secretion systems (T7SS) to export many of their important virulence proteins. The T7SS encompasses five homologous secretion systems (ESX-1 to ESX-5). Most pathogenic mycobacterial species, including the human pathogen *Mycobacterium tuberculosis*, possess all five ESX systems. The ESX-1, -3, and -5 systems are important for virulence of mycobacteria but the molecular mechanisms of their secretion apparatus and the identity and activity of secreted effector proteins are not well characterized. The different ESX systems show similarities in gene composition due to their common phylogenetic origin but recent studies demonstrate mechanistic as well as functional variations between the systems. For example, the ESX-1 system is involved in lysis of the phagosomal membrane and phagosomal escape of the bacteria while the ESX-5 system is required for mycobacterial cell wall stability and host cell lysis. Mechanistically, the ESX-1 substrates show interdependence during secretion while the ESX-5 system may use a duplicated four-gene region (ESX-5a) as an accessory system for transport of a subset of proteins of the ESX-5 secretome. In the present review we will provide an overview of the molecular components of the T7SS and their function with a particular focus on the ESX-5 system.

## The type VII secretion system components

The ability of *Mycobacterium tuberculosis* (Mtb) to subvert host immune defenses is due to the secretion of multiple virulence factors via specialized secretion systems (Ligon et al., 2012; van der Woude et al., 2013; Majlessi et al., 2015). The five type VII secretion systems (T7SS) found in mycobacteria (ESX-1 to ESX-5) were most likely duplicated from the ancestral ESX-4 system (Gey van Pittius et al., 2001, 2006). Of the five ESX systems, the ESX-5 system is thought to have duplicated most recently (Gey van Pittius et al., 2001, 2006). In fact, the evolution of the ESX-5 system coincides with the emergence of slow-growing mycobacterial species (Gey van Pittius et al., 2006). It is interesting to note that most of the mycobacterial species that are important human pathogens belong to the slow-growing lineage (Cole et al., 1998). Functionally, only the ESX-1, ESX-3, and ESX-5 systems have been proven to be involved in protein secretion (Pym et al., 2002; Stanley, 2003; Abdallah et al., 2006, 2009; Siegrist et al., 2009; Daleke et al., 2012; Tufariello et al., 2016) while no evidence of active secretion in ESX-2 or ESX-4 systems has been found to date. In addition, the ESX-1, ESX-3, and ESX-5 systems are important for virulence (Pym et al., 2002; Hsu et al., 2003; Lewis et al., 2003; Stanley, 2003; Serafini et al., 2009; Siegrist et al., 2009).

The ESX systems are comprised of genes that encode: (i) the structural components of the secretion system such as the putative channel protein (EccD), conserved membrane proteins (EccB and EccC), and AAA+ ATPase (EccA), (ii) Mycosin (MycP) which has homology to subtilisin-like proteases, and (iii) two secreted Esx proteins. Additionally, all ESX systems, except ESX-4, also contain genes encoding members of the PE/PPE protein family which derives its name from the Pro-Glu (PE) and Pro-Pro-Glu (PPE) motifs found in the N-terminus of these proteins (Abdallah et al., 2007; Sampson, 2011; Kunnath-Velayudhan and Porcelli, 2013; Houben et al., 2014; Majlessi et al., 2015).

**Figure 1:**
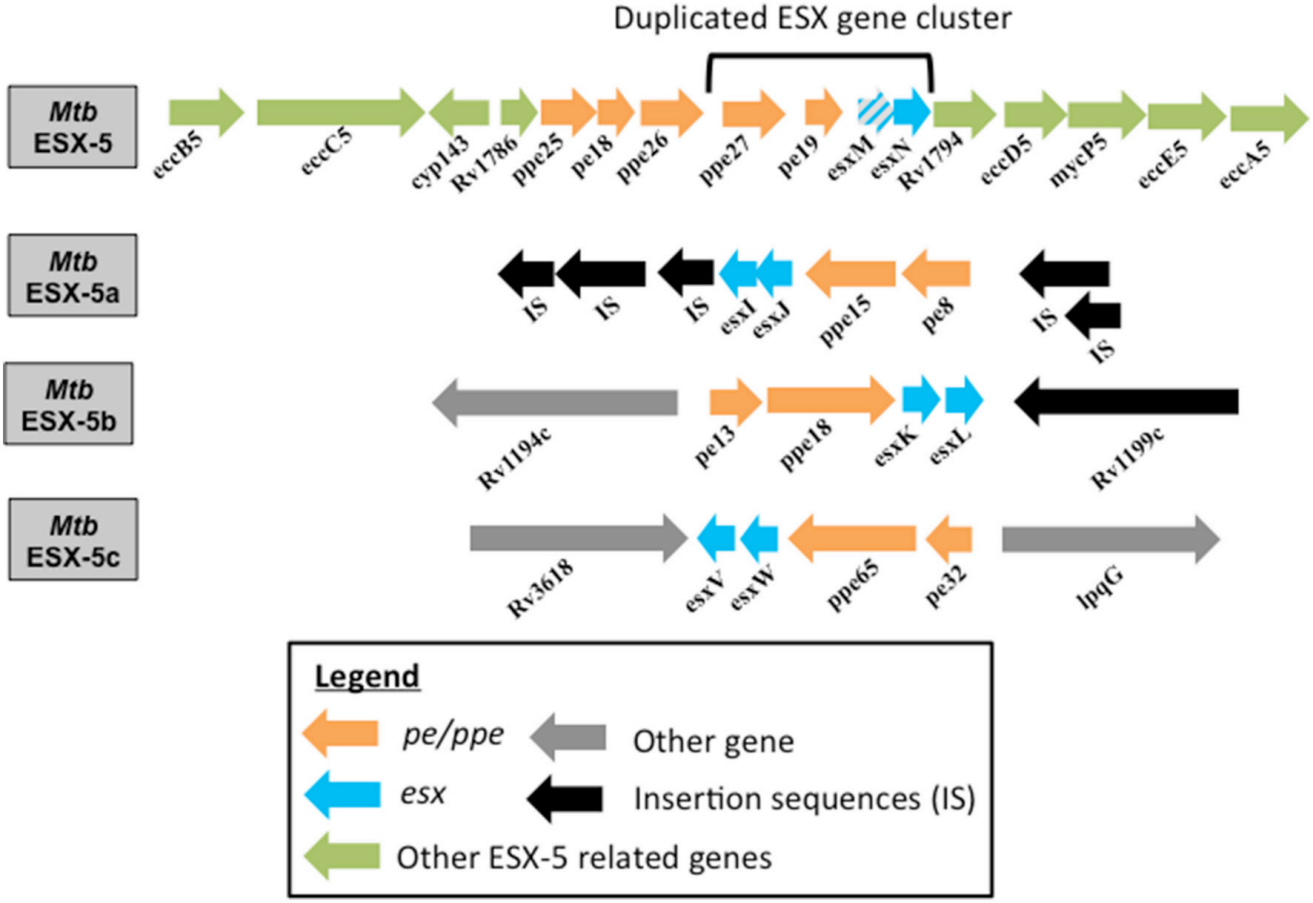
**Genome organization of ESX-5 and the duplicated ***esx*** gene clusters in Mtb**. Genome organization of the Mtb parent ESX-5 locus and the three duplicated *esx* gene cluster regions, namely ESX-5a (Rv1037c-Rv1049c), ESX-5b (Rv1195-Rv1198), and ESX-5c (Rv3619c-Rv3622c) are shown. *EsxM* is a pseudogene.

Each of the ESX systems has a pair of *esx* genes that encode for proteins belonging to the WXG100 family (Pallen, 2002). Even though the Esx proteins form an integral part of the T7SS, paralogs of these genes pairs are also found outside of the ESX loci (Gey van Pittius et al., 2006). The *pe/ppe* gene pairs are found: (1) adjacent to the *esx* gene cluster within the ESX loci, (2) adjacent to the paralogous *esx* gene pairs forming a four-gene region, or (3) isolated in the genome (Figure 1). The *pe/ppe* gene family has evolved around the time of ESX-1 duplication and thus there are no *pe/ppe* genes found associated with the ESX-4 system, since it precedes the ESX-1 system (Gey van Pittius et al., 2006). Over time, the PE/PPE protein family expanded and now accounts for about 10% of Mtb's coding potential (Cole et al., 1998; Gey van Pittius et al., 2006; Akhter et al., 2012).

## ESX-1 secretion system

The ESX-1 system was the first of the T7SS to be identified and is responsible for the secretion of EsxA and EsxB (Stanley, 2003). The tuberculosis live vaccine strain, *Mycobacterium bovis* BCG has a deletion of a 9.5 Kbp genomic stretch called the region of difference 1 (RD1) that corresponds to the ESX-1 secretion system (Behr et al., 1999). Upon reconstitution of the RD1 locus in BCG, studies demonstrated an increase in virulence in the mouse model of tuberculosis (Pym et al., 2002, 2003). These and many subsequent studies served to confirm the importance of the ESX-1 system and its substrates in Mtb virulence (Hsu et al., 2003; Lewis et al., 2003; Stanley, 2003; Fortune et al., 2005). In Mm, the ESX-1 system is required for persistence within macrophages, cell-to-cell spread and virulence in the zebrafish model (Gao et al., 2004). The ESX-1 system is involved in phagosomal membrane permeabilization and escape of Mtb from the phagosome (Stanley and Cox, 2013). This membrane permeabilization occurs early during infection and allows Mtb to manipulate various host signaling pathways (Stanley, 2003; Stanley et al., 2007; Xu et al., 2007; Welin et al., 2011; Manzanillo et al., 2012; Romagnoli et al., 2012; Shah et al., 2013). In addition to the secretion of EsxA and EsxB, the ESX-1 system is also required for the secretion of a number of *esp* (ESX-1 secretion-associated protein) encoded gene products such as EspA-C, EspE, and EspJ (McLaughlin et al., 2007; Raghavan et al., 2008; Carlsson et al., 2009; Champion et al., 2009; Singh et al., 2015). One striking characteristic of the ESX-1 system is the co-dependent nature of secretion of its substrates (Stanley, 2003; Guinn et al., 2004; Fortune et al., 2005). Since EsxA and EsxB form heterodimers, it is understandable that they require each other for secretion (Renshaw et al., 2005). However, for example, the secretion of EspA and EspC are also dependent on EsxA/EsxB secretion and vice versa (Fortune et al., 2005; MacGurn et al., 2005). Similarly, in Mm it was shown that secretion of EsxB and EspE is co-dependent (Carlsson et al., 2009). This property of co-dependency seems to be confined to the Esx and Esp proteins of the ESX-1 system, since there is no such evidence for this phenomenon in the ESX-5 system (Sampson, 2011; Houben et al., 2014).

## ESX-5 secretion system

**Figure 2:**
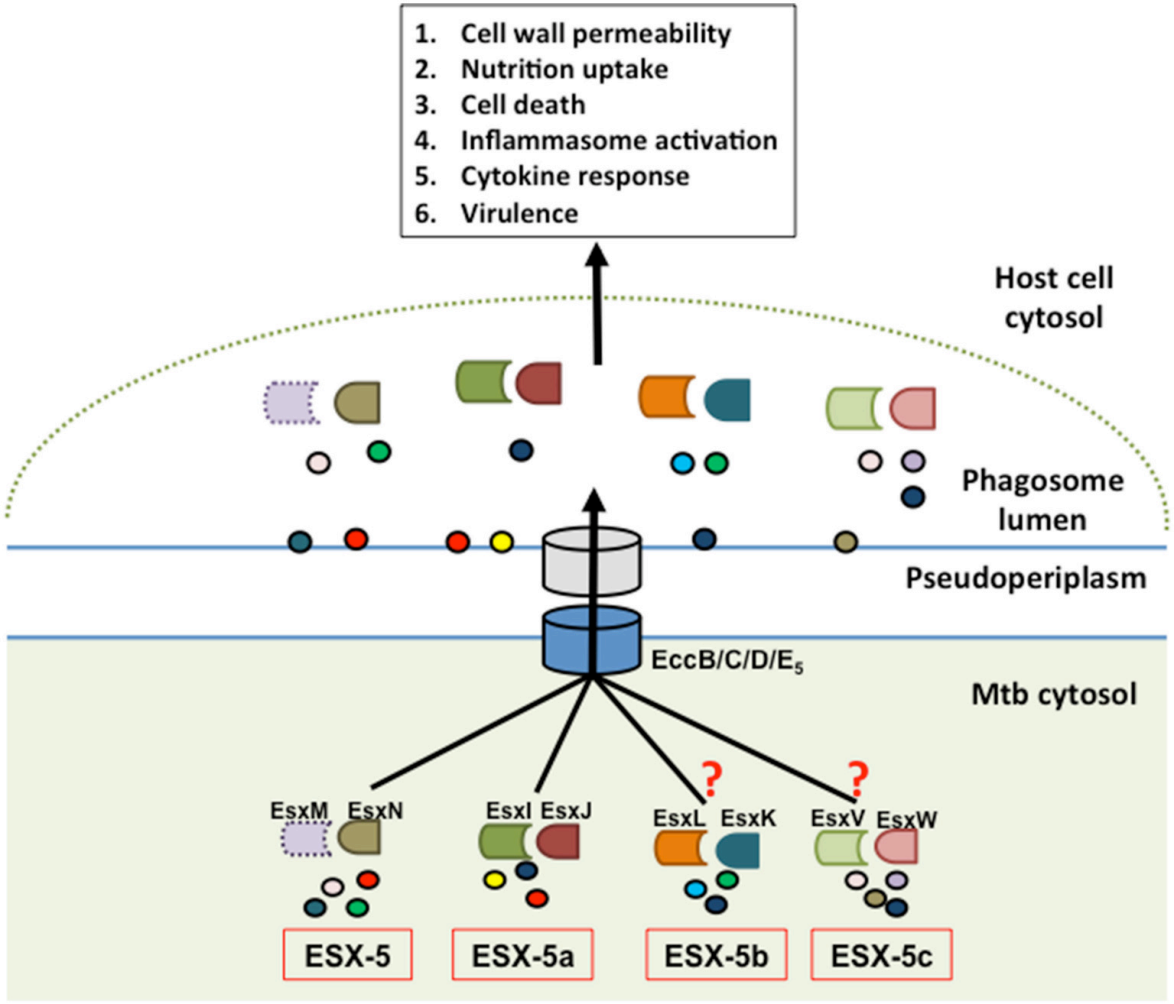
**Model of ESX-5-dependent secretion mechanism**. The hypothetical model depicts the parent ESX-5 Esx protein pair (EsxM/N) and cognate duplicate paralogs from ESX-5a (EsxI/J), ESX-5b (EsxK/L), and ESX-5c (EsxV/W) regions as having a role in substrate selection via the core ESX-5 secretion machinery and that these secreted effectors in turn manipulate various host defense processes. The EsxM protein is shown in dotted outline to indicate that it is a pseudogene. The experimental findings supporting a role of ESX-5a were extrapolated for ESX-5b and ESX-5c, which is indicated by the question marks. The circles represent putative secretion substrates associated with each of these systems. Some of these substrates may be shared between the different paralogs.

The ESX-5 secretion system is the most recently evolved of the T7SS (Gey van Pittius et al., 2001) and is activated under phosphate limiting conditions at the transcriptional level via the SenX3/RegX3 two-component system (Elliott and Tischler, 2016) to execute a multitude of functions (Figure 2). Indeed, the ESX-5 system mediates the secretion of the majority of PE/PPE proteins (Abdallah et al., 2009). A subset of the secreted proteins will remain in the cell envelope where they are important for modifying the cell permeability and uptake of hydrophobic carbon sources (Ates et al., 2015). In Mm, the ESX-5 system is also required to reduce pro-inflammatory cytokine secretion by macrophages (Abdallah et al., 2008). Furthermore, both the Mm and Mtb ESX-5 systems are required for the activation of the host cell inflammasome and consequently IL-1β secretion (Abdallah et al., 2008, 2011). Additionally, the ESX-5 system induces a caspase-independent form of cell death in macrophages after phagosomal escape of the bacteria. This allows the bacteria to exit the cells and infect neighboring cells (Abdallah et al., 2011). While evaluating the effects of ESX-5 on virulence, one study found that an Mm ESX-5 transposon insertion mutant was hypervirulent in adult zebrafish (Weerdenburg et al., 2012). However, more recent studies conducted, demonstrate that Mtb ESX-5 deletion mutants are attenuated (Bottai et al., 2012; Sayes et al., 2012). The discrepancy between the two results could be due to inherent differences between the functions of the ESX-5 systems in the two mycobacterial species Mm and Mtb. Supporting this argument is the fact that an Mm ESX-5 mutant was found to be deficient in the secretion of numerous PE_PGRS proteins (a PE sub-family) but minimal deficiency was evident in various Mtb ESX-5 mutants tested (Bottai et al., 2012). Interestingly, these findings by Bottai et al. have been contradicted by another group that demonstrates the secretion deficiency of PE_PGRS proteins in Mtb ESX-5 mutants (Houben et al., 2012). However, it is important to note that the Mtb ESX-5 mutants generated by Bottai et al. are in the H37Rv Mtb strain background while the latter study was conducted using transposon mutants in the CDC1551 Mtb strain, which may explain the discrepancy in findings. Furthermore, the reconstitution of the entire ESX-5 region in an Mm ESX-5 deletion strain with the Mtb ESX-5 genes was not sufficient to completely restore the secretion of PE_PGRS proteins (Ates et al., 2015). Therefore, these studies reveal some functional differences in ESX-5-mediated protein secretion between these two mycobacterial species. This could reflect a fundamental difference between Mtb and Mm with the former being highly adapted to humans whereas Mm infects a large variety of species and hence may need a larger repertoire of virulence factors in order to adapt to the diverse hosts as suggested in a recent publication (Weerdenburg et al., 2015). The importance of ESX-5 protein secretion is further highlighted by the discovery that PE/PPE proteins are a major target of the host acquired immune response as revealed by performing immunoproteome screens reviewed in Kunnath-Velayudhan and Porcelli (2013).

## Role of a duplicated *esx* gene cluster in ESX-5 function

A four-gene region (*esx* gene cluster) containing the two *esx* genes and the flanking *pe/ppe* gene pair of the ESX-5 system has undergone three duplication events (Gey van Pittius et al., 2006). These paralogous regions are found in locations of the Mtb genome distant from the ESX-5 region but their function is unknown (Gey van Pittius et al., 2006). Here they will be referred to as ESX-5a (*Rv1038c-Rv1041c*), ESX-5b (*Rv1195-Rv1198*), and ESX-5c (*Rv3619c- Rv3622c*) as shown in Figure 1. The importance of the ESX-5a region for protein secretion was investigated via a proteomics approach and revealed that besides the impact on secretion of some PE/PPE proteins, two novel proteins, alanine L-dehydrogenase (ALD) and superoxide dismutase A (SodA) require a functional ESX-5a region for their secretion (Shah et al., 2015). Since ESX-5a is duplicated from the ESX-5 system and a previous report shows SodA to be a substrate of the SecA2 system (Braunstein et al., 2003), the culture filtrates (CF) of the Mm *secA2* mutant, and a transposon mutant of a component of the core ESX-5 secretory machinery (*eccA*_5_*::Tn*) were tested for SodA and ALD secretion. The ESX-5 mutant was found to be defective in the secretion of both SodA and ALD while the SodA protein could be detected in the CF of the *secA2* mutant. This indicates that ALD and SodA are secreted via the ESX-5 core secretion system but require the accessory ESX-5a proteins (Shah et al., 2015).

These results suggest that the ESX-5a region is responsible for secretion of a subset of substrates via the ESX-5 system and can therefore be considered an accessory to the parent ESX-5 system (Shah et al., 2015). This is also supported by the fact that ESX-5a lacks genes encoding a functional secretion apparatus (e.g., channel protein) and hence requires interaction with a secretion system. Consequently, we hypothesize that the other two paralogs, ESX-5b, and ESX-5c, may also act as accessory systems that aid in the secretion of a subset of proteins via the parent ESX-5 system (Figure 2). Furthermore, the parent ESX-5 *esx* gene cluster has a similar chaperone function since deletion of *esxM/N* did not abolish secretion of all ESX-5 substrates as opposed to deletion of the gene encoding the putative ESX-5 channel protein, EccD_5_ in Mtb (Bottai et al., 2012).

In conclusion, we propose a model by which the three accessory systems (ESX-5a, b, and c) along with the parent *esx* gene cluster are each responsible for the secretion of a subset of the total ESX-5 secretome and in doing so, contribute to the overall function of the ESX-5 system (Figure 2). Consequently, for the ESX-5 system the deletion of one accessory region does not abolish the secretory functions of the other systems although there could be some overlap for a few of the secreted proteins. This mechanism is in sharp contrast to that of the ESX-1 system, whose substrates are secreted in a co-dependent fashion (Stanley, 2003; Guinn et al., 2004; Fortune et al., 2005). The reason for such division of labor among the parent ESX-5 and accessory ESX-5 regions could be due to the sheer volume of PE/PPE proteins that need to be exported (the *pe/ppe* genes make up about 10% of the total Mtb genome) and/or the necessity to differentially regulate the subsets of PE/PPE proteins that are being secreted during the course of an infection (Abdallah et al., 2009; van der Woude et al., 2013). The latter point was supported by further characterization of the ESX-5a deletion mutant showing that the ESX-5a-dependent substrates are involved in inflammasome activation but do not have any role in host cell death mediation which is reduced in ESX-5 deficient mutants (Shah et al., 2015). These results suggest that by deleting each of the accessory ESX-5 regions and by characterizing their phenotypes and secretion defects, we may able to attribute functions to a smaller subset of the vast ESX-5 secretome. For example, the ESX-5a substrates are shown to be involved in activating the host cell inflammasome but not cell death. Therefore, the substrates from other accessory regions could be responsible for the host cell death phenotype. Thus, this approach may help assign functions to a smaller group of PE/PPE proteins and should facilitate the discovery of specific effector functions for individual ESX-5-secreted proteins.

## How is the substrate selection by the ESX-5 system mediated?

The first evidence for a specific peptide signal in T7SS substrates was discovered by deleting the C-terminal 7 amino acids of EsxB and EspC, substrates of the ESX-1 system(Champion et al., 2006, 2009; Champion and Cox, 2007). The C-terminal sequence of EsxB and EspC are necessary to mediate ESX-1-dependent secretion and to allow for the interaction with components of the core T7SS secretion pore, EccC_1_, or EccA_1_, respectively (Champion et al., 2006, 2009). A general secretion signal, YxxxD/E, was first identified in the C-terminus of a member of the PE-protein family which are mainly secreted via the ESX-5 system but in some cases also via the ESX-1 system (Daleke et al., 2012). Interestingly, this conserved motif is also present adjacent to the 7 C-terminal amino acids of EsxB and EspC and mutation of the tyrosine in that motif reduces secretion of the proteins (Daleke et al., 2012). Overall, all of the dimeric substrates (Esx and PE/PPE proteins) of T7SS secretion systems share many structural features and the presence of the YxxxD/E motif in one of the proteins of the heterodimers (Houben et al., 2014; Majlessi et al., 2015). Furthermore, protein structure analysis and functional protein–protein interaction studies demonstrated that the EspG_5_ protein interacts with the N-terminus of the PPE protein of the PPE/PE-heterodimer without binding the PE protein or covering the C-terminal regions nor affecting the structure of the PPE/PE dimer in any way (Daleke et al., 2011; Ekiert and Cox, 2014; Korotkova et al., 2014). In contrast to the PPE/PE proteins, EspG_5_ is not secreted. Both groups propose a similar model in which the EspG_5_ protein serves as a signal recognition particle in analogy to conserved secretion pathways that transports newly synthesized PPE/PE complexes to the ESX-5 system at the bacterial membrane, at which point the cytosolic ATPase EccA helps to dissociated EspG_5_ from the PPE/PE-dimer (Ekiert and Cox, 2014; Korotkova et al., 2014). Ekiert and Cox furthermore propose the hypothesis that the free C-terminus of the PPE/PE/EspG_5_ complex may serve as a binding site for cargo proteins that will be secreted via the ESX-5 system (Ekiert and Cox, 2014). In the light of our data of the modular organization of the ESX-5 system in regard to its usage of duplicated *esx* gene clusters, it is compelling to further hypothesize that the pair of Esx proteins also play an important part by associating with the PPE/PE dimer and assisting in cargo selection. This could explain why the EsxA/B proteins are essential for all the secretion via the ESX-1 system but the paralog EsxM/N pair are not for ESX-5-dependent substrates because of the duplicated *esx* gene clusters (Shah et al., 2015).

## Conclusions

The ESX-1 system is the best-characterized T7SS and has therefore served as a blueprint for our understanding of the molecular mechanisms of T7SS. The ESX-5 secretion system is involved in the export of the vast majority of PE/PPE proteins to the cell surface and culture supernatant. We provide evidence that at least one of the four-gene, *esx* gene cluster duplicated from the ESX-5 system acts as an accessory region that is required for the secretion of a subset of the ESX-5 secretome. The use of a paralogous region for substrate compartmentalization by the ESX-5 system is in sharp contrast to the co-dependent mechanisms of ESX-1 substrate secretion and its absence of duplicated regions. Here we propose a refined model for the molecular mechanism of ESX-5-dependent secretion. Future studies should characterize the other ESX-5 duplicated regions and confirm their role in the ESX-5 secretion system and consequently test our hypothetical model for secretion of ESX-5-dependent substrates. A systematic characterization of the secretomes and associated phenotypes for all three putative ESX-5 accessory regions and the parent ESX-5 *esx* gene cluster will also help in assigning a specific function to a smaller subset of PE/PPE proteins. This will facilitate the discovery of a specific function for some of the PE/PPE proteins because of this smaller pool of candidate proteins. The documented importance of ESX-5 for virulence of Mtb makes the discovery of the effector proteins highly relevant for drug and vaccine development.

## Author contributions

All authors listed, have made substantial, direct, and intellectual contribution to the work, and approved it for publication.

### Conflict of interest statement

The authors declare that the research was conducted in the absence of any commercial or financial relationships that could be construed as a potential conflict of interest.
